# Oxidative stress and acute pancreatitis (Review)

**DOI:** 10.3892/br.2024.1812

**Published:** 2024-06-27

**Authors:** Yongxia Cai, Feng Yang, Xizhu Huang

**Affiliations:** 1 Department of Emergency Medicine, Sir Run Run Shaw Hospital, Zhejiang University School of Medicine, Hangzhou, Zhejiang 310016, P.R. China; 2 Department of Emergency Medicine, The First People's Hospital of Wuyi County, Jinhua, Zhejiang 321200, P.R. China

**Keywords:** acute pancreatitis, oxidative stress, antioxidants, reactive oxygen species, antioxidant therapy

## Abstract

Acute pancreatitis (AP) is a common inflammatory disorder of the exocrine pancreas that causes severe morbidity and mortality. Although the pathophysiology of AP is poorly understood, a substantial body of evidence suggests some critical events for this disease, such as dysregulation of digestive enzyme production, cytoplasmic vacuolization, acinar cell death, edema formation, and inflammatory cell infiltration into the pancreas. Oxidative stress plays a role in the acute inflammatory response. The present review clarified the role of oxidative stress in the occurrence and development of AP by introducing oxidative stress to disrupt cellular Ca2^+^ balance and stimulating transcription factor activation and excessive release of inflammatory mediators for the application of antioxidant adjuvant therapy in the treatment of AP.

## 1. Introduction

Acute pancreatitis (AP) is a potentially fatal inflammatory disease, which, in its severe form, is associated with a mortality rate of 15-25% and often caused by pancreatic enzyme activation from various etiologies and is characterized by local inflammatory reactions in the pancreas (1-4). The etiology of AP includes mainly gallstones and excessive alcohol consumption (5-7). Patients with more severe conditions can develop systemic inflammatory response syndrome (SIRS), which may be accompanied by organ dysfunction (6-8). Clinically, it is characterized by acute upper abdominal pain and elevated levels of blood amylase or lipase (6,7). AP has a rapid onset and changes rapidly. When accompanied by multiple organ dysfunction and local complications of the pancreas, the mortality rate significantly increases (9,10). At present, the pathogenesis of AP is not very clear.

In the early stages of AP, various stimulating factors cause premature activation of trypsin in pancreatic acinar cells (1,11). This process involves various pathogenesis mechanisms, such as pathological calcium signaling in pancreatic acinar cells, changes in pH, coexistence and autophagy, the cleavage of trypsinogen by the lysosomal hydrolytic enzyme tissue protease B to trypsin, and decreased activity of the pancreatic acinar cell trypsin inhibitor (11-13). Once trypsin is activated, various damaging pancreatic digestive enzymes become active, and the pancreas and adjacent tissues undergo self-digestion, causing local inflammation in the pancreas and the secretion of a large amount of TNF-α. Inflammatory cytokines can cause necrosis of pancreatic acinar cells and enter the bloodstream, promoting the secretion of inflammatory cytokines such as IL-1, IL-6 and IL-8, resulting in a waterfall effect (9,14,15). The activation of endoplasmic reticulum stress, the unfolded protein response, autophagy, oxidative stress, lysosomal and mitochondrial dysfunction, and signaling pathways are all mechanisms underlying the pathogenesis of AP (10,16-19).

Oxidative stress is a stress state that causes an imbalance between oxidative and antioxidative effects in the body, leading to inflammatory infiltration of neutrophils, increased protease secretion and the production of numerous oxidative intermediates, causing oxidative damage and interfering with the metabolic activities of normal organs (18,20,21). There are two types of antioxidant systems in the body. One is the enzyme antioxidant system, which includes superoxide dismutase, catalase and glutathione peroxidase (22,23). The other is the nonenzymatic antioxidant system, which includes ertenionine, vitamin C, vitamin E, glutathione and melatonin (24,25). When human and animal cells are stimulated by nitrogen and nitrogen compounds, as well as calcium and pathogens, the balance between oxidation and antioxidant systems is disrupted, thus promoting the increased production and accumulation of reactive oxygen species (ROS) in the cell and eventually leading to an oxidative stress response (20,26). The redox balance of body cells determines the longevity of cells. In the past few decades, numerous studies have emphasized the role of oxidative stress in the acute inflammatory response (27-29). Currently, oxidative stress is considered not only a key mediator associated with early local events of AP but also a key mediator associated with systemic inflammatory response syndrome (20,30). Oxidative stress is involved in the occurrence and development of pancreatitis. The different stages and concentrations of ROS have different effects. Relevant reports suggest that ROS are beneficial for cell apoptosis during the acute phase, while reducing necrosis and preventing severe pancreatic damage (18,20,24). ROS appear to protect acinar cells, but high concentrations can lead to pancreatic damage. The present review examines the role of oxidative stress in the development of AP.

## 2. Reactive oxygen species

ROS are free radicals containing oxygen atoms, including superoxide anions, hydroxyl radicals, hydrogen peroxide, hypochlorous acid and singlet oxygen (18,31). They have complex signaling functions and play important roles in the development of inflammatory diseases. Oxidative stress occurs when there is an imbalance between the antioxidant defense system and the production of ROS (32). ROS can quickly combine with nitric oxide (NO) to form reactive nitrogen species (RNS), decreasing the endogenous antioxidant protection ability of the body and participating in the occurrence and development of AP (20,21).

Biofilms contain polyunsaturated fatty acids, which are highly susceptible to ROS attack and undergo lipid peroxidation. Lipid peroxidation is a chain reaction process of unsaturated fatty acid oxidation degradation that has three stages: Initiation, extension, and termination (33). The extension stage of lipid peroxidation produces various free radicals, such as lipid peroxidation free radicals, lipid oxygen free radicals and lipid free radicals (34). The termination stage involves the production of various small molecule products, such as malondialdehyde (MDA), which can cause damage to various cellular functions, which is closely related to the occurrence and development of various diseases and is an important marker of the early severity of disease (35,36). It is well known that ROS serve numerous important biological functions, including the regulation of redox-sensitive transcription factors, redox-sensitive signal transduction pathways and direct interactions with various molecules (18,20). The large amount of ROS produced by oxidative stress can cause cell necrosis or apoptosis through different pathways. At present, it has been confirmed that the oxidative stress response is related to various diseases, and there is notable research in cardiology, neurology and endocrinology, as well as in other fields (37). Vasopressin can protect the myocardium by antioxidation and inhibition of mitochondrial permeability, reducing myocardial ischemia-reperfusion injury (38). Taurine can prevent and treat vascular dysfunction by reducing the vascular oxidative stress response (39). Vitamin E has been proven to reduce oxidative stress responses in the liver and kidneys, thereby playing a protective role (40). Mangiferin can inhibit nuclear factor κB (NF-κB) and increase catalase activity to protect cells and has therapeutic significance in clinical practice (41,42). Unsaturated fatty acids can exert antioxidant effects on cell membranes. Dietary flavonoids and their sources have a protective effect on cardiovascular disease through antioxidant activity (43). Some traditional Chinese medicines have also been proven to have antioxidant stress response effects. Studies have shown that Gegenqinlian decoction can significantly increase the activity of superoxide dismutase and reduce the activity of malondialdehyde, nitric oxide synthase (NOS), tumor necrosis factor, interleukin, and inflammatory cytokines in the colon, thereby regulating the balance between oxidants and antioxidants and having a protective effect on ulcerative colitis (44). Schisandrin B has been revealed to activate the Nrf2/ARE pathway to alleviate cisplatin-induced oxidative stress damage in renal cells (31).

Research has shown that the large amount of ROS produced by oxidative stress can cause cell necrosis or apoptosis through different pathways: i) Mitochondrial dysfunction and disruption of lipid and lysosomal membranes (10); ii) disruption of the intracellular environment of the cytoplasm, causing intracellular Ca2^+^ overload (45); iii) increased lipid peroxidation of the cell membrane caused by free radicals which reduces membrane fluidity, and fluidity enhances permeability, and increases extracellular calcium ion influx, leading to cell death (46,47); iv) activation of the apoptosis-related genes, Bax and p53, on the mitochondrial membrane of pancreatic acinar cells, as well as the release of cytochrome *c* (48); v) activation of the oxidative stress-sensitive transcription nuclear factor factor κB (NF-κB) (46); vi) activation of the c-Jun amino terminal kinase (JNK) and p38 mitogen-activated protein kinase (p38 MAPK) pathways (49); vii) activation of CD95 receptors and in turn cell apoptosis; and viii) production of inflammatory mediators (50). Changes in signaling pathways are controlled by the level of the oxidative stress response, and the degree of oxidation-reduction plays a more important role in pathological and physiological processes than does the accumulation of oxidative damage (30). In addition, changes in the oxidative stress response and redox degree play different roles in pathological and physiological processes involving different cellular components, and their combined effect is stronger than any individual effect. The oxidative stress response and redox reactions can produce different physiological and pathological outcomes in different organs, tissues and cells.

The antioxidant system includes *in vivo* antioxidants such as vitamin C, selenium, and nicotinamide adenine dinucleotide, as well as enzymes such as superoxide dismutase (SOD), peroxidase, catalase, glutathione peroxidase (GPx) and glutathione reductase, and antioxidant mechanisms such as cellular autophagy (18). Research has shown that GPx, glutathione and metallothionein play the first line of defense against oxidative stress and antioxidant imbalance during the AP process (51). Glutathione and glutathione disulfide are in equilibrium, and the ratio between the two is a reliable indicator of oxidative stress, as it reflects the balance between antioxidant status and the pro-oxidant response in cells. Mitochondria contain small molecule antioxidants such as ascorbic acid, glutathione and vitamin E. Both ascorbic acid and glutathione are actively transported to mitochondria, thereby preventing oxidative damage to mitochondrial DNA (52). Melatonin is a product of the pineal gland that is released from the intestinal mucosa in response to food intake. Specific receptors for melatonin have been detected in numerous gastrointestinal tissues, including the pancreas. Melatonin and its precursor L-tryptophan can alleviate the severity of AP and protect pancreatic gland tissue from damage caused by acute inflammatory reactions (53). Previous research has shown that nicotinamide adenine dinucleotide phosphate oxidase inhibitors can significantly reduce AP-related inflammation and oxidative stress parameters (54). Coenzyme Q10 is the only endogenous lipid soluble antioxidant with favorable antioxidant and anti-inflammatory effects (55). It is the most common type of coenzyme Q in human tissues. Autophagy is the main metabolic process in the body. Autophagy plays an important role in clearing defective or harmful cytoplasm, organelles, denatured proteins and lipids, and recycling their components to meet energy and biological needs. Under stress, autophagy plays a crucial role in clearing free radicals and maintaining protective cell function (18,19).

## 3. Oxidative stress and acute pancreatitis

AP is a sterile inflammation of the pancreas that can initially progress from mild self-limiting local inflammation to life-threatening severe AP (SAP) as the disease progresses (22). A series of local and systemic complications may occur, and in severe cases, multiple organ dysfunction syndrome (MODS) may even occur (12). ROS act as both signaling molecules and inflammatory mediators in the early stages of AP, playing important roles in both local pancreatic injury and cellular damage to extrapancreatic organs (16,39). The acute inflammatory response begins with immune cells. Neutrophils are the first batch of cells that adhere to endothelial cells, and they begin to migrate through the vascular wall at the site of infection to engulf invading pathogens. The ROS removal system is impaired, and autophagy defects lead to an increase in oxidative stress levels in mitochondria (10,25). Oxidative stress directly affects biological molecules such as DNA, proteins and lipids, regulating gene transcription and protein expression (1). Once the stimulus persists or overwhelms, it can lead to disease progression and complications. Free radicals react with polyunsaturated fatty acids in the cell membrane to produce intermediate products such as malondialdehyde and 4-hydroxynonanal, leading to cell membrane damage and cell death (56). ROS are recognized as important causes of pancreatic cell death (18). In pancreatitis-associated MODS, ROS directly cause cellular damage and regulate redox-sensitive transcription factors and redox-sensitive signaling pathways (42). In 1995, Ward *et al* proposed that disrupting the calcium balance of acinar cells is the basis for the development of pancreatic injury. These authors suggested that various pathogenic factors of AP exacerbate the course of AP by causing excessive release of acinar calcium ions or disrupting low resting levels of calcium ions. Continuous calcium overload may activate degradable calpain, phospholipase or other enzymes and destroy proenzyme particles, inducing autophagy and/or lysosomal activation of digestive enzyme reactions (57). Cytoplasmic Ca2^+^ has been described as a key regulator of the development of AP (45,47). A large body of evidence suggests that Ca2^+^ signaling is closely related to ROS (47). The ROS produced by mitochondrial electron transport chains may interact with targets involved in the dynamic balance of cellular Ca2^+^, thereby altering their activity (45). For example, 2- and 3-inositol 1,4,5-triphosphate receptors and ryanodine receptors, which contain multiple cysteine residues, are sensitive to ROS, indicating that the regulatory effect of free radicals on Ca2^+^ release channels may affect disease progression (45). There are numerous factors that induce the formation of AP, such as caerulein over-stimulation, bile salt, nonoxidative alcohol metabolites (FAEs) and fatty acids, and these stimulating factors induce the formation of mitochondrial membrane permeability transition pores (MPTPs), which are solute channels regulated by cyclophilin D (CypD), and then lead to enzyme activation, ATP depletion and cell necrosis. ROS play a crucial role in the formation of the MPTP, which leads to cell necrosis by altering the conformation and activity of CypD (10,45,58). In addition, Armstrong *et al* reported that ROS alter mitochondrial bioenergetics independently of CypD and alter the mode of pancreatic cell death, leading to a transition from apoptosis to necrosis (59). In alcoholic AP, alcohol metabolism increases fatty acid ethyl esters (FAEEs) through its nonoxidative metabolism and leads to acetaldehyde, acetate and ROS through its oxidative metabolism. FAEEs have various harmful effects, such as disrupting Ca2^+^ homeostasis in acinar cells and activating pathological proenzyme transcription factors. Acetaldehyde enhances the function of the latter, leading to increased production of proinflammatory cytokines (60). Ethanol also increases inducible NOS (iNOS) production, which can produce NO free radicals. The mechanism underlying the induction of oxidative stress and ethanol-induced pancreatic injury is complex and may involve oxidative stress (61). The interaction between endoplasmic reticulum stress and inflammation leads to the harmful effect of ethanol on the pancreas.

Increasing evidence suggests that the interaction between oxidative stress and cytokines is associated with the development of AP, leading to uncontrolled inflammatory cascade amplification and MODS. Proinflammatory cytokines and oxidative stress mainly trigger each other by activating mitogen-activated protein kinases and NF-κB, leading to amplification of the inflammatory cascade (42,44). In addition, proinflammatory cytokines, especially TNF-α, interact with oxidative stress to form a vicious cycle in AP. An increase in TNF-α accumulation promotes the production of other inflammatory cytokines, including IL-1β and IL-6. This leads to the activation of the inflammatory cascade, resulting in damage to multiple tissues and organs. The levels of IL-1β and IL-6 have been demonstarted to be correlated with the severity of AP (27,30).

H_2_O_2_ promotes the apoptosis of acinar cells at low concentrations, while higher concentrations cause rapid necrosis of acinar cells (62). Booth *et al* reported contradictory findings in clinical trials of antioxidants. The production of ROS may constitute a protective mechanism for handling stress in pancreatic acinar cells, as bile acid-induced ROS increase cell apoptosis and reduce necrosis (63). Apoptosis can maintain the integrity of the plasma membrane, while necrotic cells release their components, thereby damaging neighboring cells and promoting inflammation (62,63). ROS cannot only be considered as playing a negative role, as they can also be beneficial. Considering the role of ROS in pancreatic pathophysiology, it should be recognized that there are multiple targets for ROS. A more cautious approach should be undertaken when dealing with ROS.

## 4. Antioxidant therapy for acute pancreatitis

**Figure 1:**
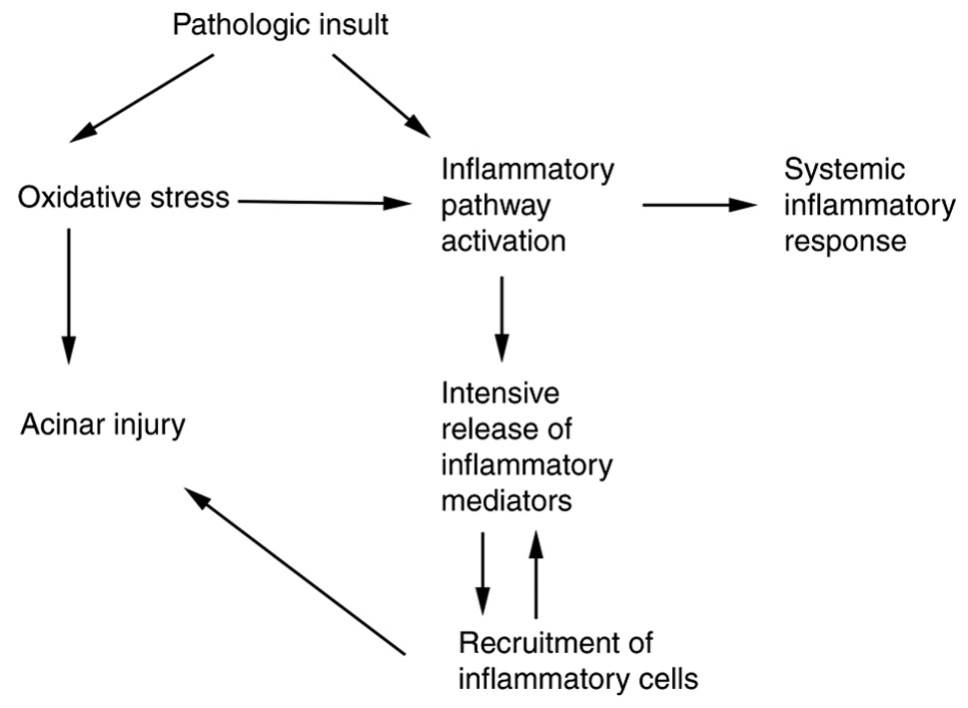
Schematic of oxidative stress in the pathophysiology of acute pancreatitis. Activation of inflammatory pathways such as NF-κB, MAPK, PI3k-Akt in the acinar cell leads to an intense inflammatory reaction responsible for local injury and the systemic inflammatory response in acute pancreatitis.

Due to the important role of oxidative stress in the pathogenesis of AP (Fig. 1), a number of studies in both humans and animal models have analyzed the association between AP and oxidative metabolism (8,22). The use of antioxidants in combination with conventional therapy may improve organ and tissue damage caused by oxidative stress. Treatment with antioxidant agents has been shown to reduce acinar cell damage and edema in several animal models. The antioxidants commonly used by the investigators are N-acetylcysteine (NAC) (64), α-tocopherol (65), β-carotin serlabo (66), selenium (67,68), melatonin (69,70), resveratrol, mitochondrial-targeted antioxidants (71), pyrrolidine dithiocarbamate (72), carnitine, traditional Chinese medicine [carvacrol (73), total saponin of *Panax notoginseng* (74)], and vitamin C (75).

NAC reduces oxidative stress parameters, serum amylase levels, and serum calcium and lactate dehydrogenase levels and reduces histopathological scores in combination with hyperbaric oxygen. The protective effects of NAC are, at least, partly due to a decrease in the production of TNF-α by acinar cells, which is concomitant with the inhibition of NF-κB(p65) nuclear translocation (64). α-Tocopherol is a phenolic antioxidant that can inhibit the autoxidation of lipids by scavenging free radicals and reacting with singlet oxygen (65). In patients with mild AP, the concentration of β-carotenoids was revealed to be significantly greater than that in patients with SAP, as β-carotenoids have antioxidant activities and are effective in protecting the human body against various oxidative stress-related diseases. β-carotenoids can increase the level of antioxidants in the body, trapping ROS and reducing oxidative damage to important biomolecules such as membrane lipids, enzymatic proteins and DNA, thereby ameliorating oxidative stress (66). Currently, the role of the micronutrient selenium is receiving increasing attention. The selenium content in the toenails of patients with SAP was revealed to be lower, and the selenium concentration in red blood cells was also revealed to be lower (67,68). In a rain frog hormone-induced AP model melatonin was demonstrated to reduce inflammation levels by regulating NF-κB, confirming its effective antioxidant and anti-inflammatory functions (69,70). Mitochondrion-targeted antioxidants have recently been shown to have cytoprotective effects, while the recently reported mitochondrion-targeted antioxidant SkQ1 scavenges ROS at nanoscale concentrations (71,76). The antioxidant pyrrolidine dithiocarbamate may inhibit NF-κB activation, thereby blocking TNF-α synthesis and thus indirectly inhibiting high mobility group protein generation and reducing pancreatic tissue damage in rats with SAP (72). The antioxidant effect of traditional Chinese medicine has improved treatment efficacy. In recent years, natural extracts or artificially synthesized antioxidants have been widely explored, and monocyclic aromatic hydrocarbons from carvacrol, which can regulate oxidative stress and reduce pancreatic cell damage, have been shown to have favorable antioxidant activity in SAP rat models. Carvacrol potentially alleviates hyperuricemia-induced oxidative stress and inflammation by regulating the ROS/NRLP3/NF-κB pathway, thereby exerting protective effects against joint degeneration (77). By upregulating the expression level of miR-181b, it can be concluded that the total saponins in *Panax notoginseng* can significantly reduce taurocholide-induced pancreatic injury and increase cell apoptosis, indicating its extensive potential in the treatment of taurocholide-induced SAP (74).

In recent years, the clinical application of vitamin C in various diseases of the body has become increasingly widespread. As the most important water-soluble antioxidant in the body, vitamin C has shown favorable clinical efficacy for burns (78), acute and chronic pain (79), severe treatment, sepsis (80), ischemia-reperfusion injury (81), blood pressure control (82), emotional improvement in hospitalized patients (83), and acute kidney disease and for reducing the mortality rate of critically ill patients (84). Du *et al* observed the clinical efficacy of high-dose vitamin C (10 g/d, daily intravenous infusion) in patients with AP. The results showed that vitamin C improved cellular antioxidant and immune abilities and exerted a favorable therapeutic effect on AP (75). Currently, clinical research on antioxidant therapy for AP mainly involves the combined application of multiple antioxidants. However, there are relatively few clinical and laboratory studies on the use of high-dose vitamin C alone for antioxidant therapy. The clinical benefits of combining vitamin C antioxidant therapy in patients with acute inflammation, traumatic stress and critical illness, warrant further research on the basic and clinical aspects of high-dose (>5 g/d) vitamin C participation in AP antioxidant therapy.

Clinical studies have shown that the consumption of serum antioxidants during the course of AP is positively correlated with the severity of the condition (85). However, there is still controversy over whether antioxidant therapy should be supplemented during SAP treatment. In an L-arginine-induced rat AP model, Handharm *et al* used a combination of intravenous infusion of NAC, selenium and vitamin C for antioxidant therapy. Early active antioxidant therapy can significantly reduce damage to the pancreas and extrapancreatic organs (86). However, the clinical study of Siriwardena *et al*, which included 43 patients with SAP, and where antioxidant therapy was administered within 7 days after patient admission (combined use of NAC, selenium and vitamin C) indicated that patients did not have any clinical benefits. Thus, it appears that the effectiveness and rationality of antioxidant use as adjunctive therapy for SAP have been called into question (87).

## 5. Conclusion

Oxidative stress disrupts the activation of cellular Ca2^+^ and the excessive release of inflammatory mediators. As the disease progresses, various factors accelerate the production of ROS, further disrupting the balance between the oxidative and antioxidant systems and forming a vicious cycle. This promotes the occurrence of oxidative stress at various stages of AP. In recent years, the role of antioxidant adjuvant therapy in the comprehensive treatment of AP and other diseases has gradually attracted the attention of clinical physicians, but related research remains in its early stages. A large amount of clinical and basic research still needs to be carried out. Future studies are required to clarify the link between the different concentrations of antioxidants and the severity of AP. In addition, the dynamic changes of oxidative stress in the course of AP can be eludicated by monitoring the changes in indicators such as free radical content and antioxidant enzyme activity in the body of a patient, thereby providing a basis for evaluating the condition and adjusting treatment plans. In summary, the relationship between oxidative stress and AP is a challenging and promising research field. Through continuous in-depth research and exploration, more precise and effective strategies for the treatment and prevention of AP may be provided, bringing better quality of life and health well-being to patients.

A limitation in the present review, is that only the relationship between oxidative stress and AP was explored. Therefore, the relationship between chronic pancreatitis and oxidative stress should be investigated in the future.

## Data Availability

Not applicable.
